# Supplementary material for: Resolving the full spectrum of human genome variation using Linked-Reads
Source: Genome Res. 2019 Apr;29(4):635–45. doi: 10.1101/gr.234443.118 (PMC6442396; doi:10.1101/gr.234443.118)
Supplement: Supplemental Material [file supp_gr.234443.118_Supplemental_longranger-2.2.zip › longranger-2.2.tar.bz2!/lr2.2-public/lib/stan/cmdstan/stan/doc/api/html/agrad_2rev_2functions_2log__falling__factorial_8hpp_source.html]

Stan: src/stan/agrad/rev/functions/log\_falling\_factorial.hpp Source File


|  |  |
| --- | --- |
|  | Stan  2.5.0  probability, sampling & optimization |


- Main Page
- Related Pages
- Namespaces
- Classes
- Files

- File List
- File Members

All Classes Namespaces Files Functions Variables Typedefs Enumerations Enumerator Friends Macros Pages

- src
- stan
- agrad
- rev
- functions

log\_falling\_factorial.hpp

Go to the documentation of this file.

1 #ifndef STAN\_\_AGRAD\_\_REV\_\_FUNCTIONS\_\_LOG\_FALLING\_FACTORIAL\_HPP

2 #define STAN\_\_AGRAD\_\_REV\_\_FUNCTIONS\_\_LOG\_FALLING\_FACTORIAL\_HPP

3

4 #include <stan/agrad/rev/var.hpp>

5 #include <stan/agrad/rev/internal/vd\_vari.hpp>

6 #include <stan/agrad/rev/internal/dv\_vari.hpp>

7 #include <stan/agrad/rev/internal/vv\_vari.hpp>

8 #include <stan/math/functions/log\_falling\_factorial.hpp>

9 #include <boost/math/special\_functions/digamma.hpp>

10 #include <boost/math/special\_functions/fpclassify.hpp>

11

12 namespace stan {

13  namespace agrad {

14

15  namespace {

16

17  class log\_falling\_factorial\_vv\_vari : public op\_vv\_vari {

18  public:

19  log\_falling\_factorial\_vv\_vari(vari\* avi, vari\* bvi) :

20  op\_vv\_vari(stan::math::log\_falling\_factorial(avi->val\_, bvi->val\_), avi, bvi) {

21  }

22  void chain() {

23  if (unlikely(boost::math::isnan(avi\_->val\_)

24  || boost::math::isnan(bvi\_->val\_))) {

25  avi\_->adj\_ = std::numeric\_limits<double>::quiet\_NaN();

26  bvi\_->adj\_ = std::numeric\_limits<double>::quiet\_NaN();

27  } else {

28  avi\_->adj\_ += adj\_ \* boost::math::digamma(avi\_->val\_ + 1);

29  bvi\_->adj\_ += adj\_ \* -boost::math::digamma(bvi\_->val\_ + 1);

30  }

31  }

32  };

33

34  class log\_falling\_factorial\_vd\_vari : public op\_vd\_vari {

35  public:

36  log\_falling\_factorial\_vd\_vari(vari\* avi, double b) :

37  op\_vd\_vari(stan::math::log\_falling\_factorial(avi->val\_, b), avi, b) {

38  }

39  void chain() {

40  if (unlikely(boost::math::isnan(avi\_->val\_)

41  || boost::math::isnan(bd\_)))

42  avi\_->adj\_ = std::numeric\_limits<double>::quiet\_NaN();

43  else

44  avi\_->adj\_ += adj\_ \* boost::math::digamma(avi\_->val\_ + 1);

45  }

46  };

47

48  class log\_falling\_factorial\_dv\_vari : public op\_dv\_vari {

49  public:

50  log\_falling\_factorial\_dv\_vari(double a, vari\* bvi) :

51  op\_dv\_vari(stan::math::log\_falling\_factorial(a, bvi->val\_), a, bvi) {

52  }

53  void chain() {

54  if (unlikely(boost::math::isnan(ad\_)

55  || boost::math::isnan(bvi\_->val\_)))

56  bvi\_->adj\_ = std::numeric\_limits<double>::quiet\_NaN();

57  else

58  bvi\_->adj\_ += adj\_ \* -boost::math::digamma(bvi\_->val\_ + 1);

59  }

60  };

61  }

62

63  inline var log\_falling\_factorial(const var& a,

64  const double& b) {

65  return var(new log\_falling\_factorial\_vd\_vari(a.vi\_, b));

66  }

67

68  inline var log\_falling\_factorial(const var& a,

69  const var& b) {

70  return var(new log\_falling\_factorial\_vv\_vari(a.vi\_, b.vi\_));

71  }

72

73  inline var log\_falling\_factorial(const double& a,

74  const var& b) {

75  return var(new log\_falling\_factorial\_dv\_vari(a, b.vi\_));

76  }

77  }

78 }

79 #endif

boost::math::isnan

bool isnan(const stan::agrad::var &v)

Checks if the given number is NaN.

**Definition:** boost\_fpclassify.hpp:69

dv\_vari.hpp

vd\_vari.hpp

stan::agrad::var::vi\_

vari \* vi\_

Pointer to the implementation of this variable.

**Definition:** var.hpp:40

stan::agrad::log\_falling\_factorial

fvar< T > log\_falling\_factorial(const fvar< T > &x, const fvar< T > &n)

**Definition:** log\_falling\_factorial.hpp:15

unlikely

#define unlikely(x)

**Definition:** likely.hpp:9

log\_falling\_factorial.hpp

var.hpp

stan::agrad::var

Independent (input) and dependent (output) variables for gradients.

**Definition:** var.hpp:27

stan::agrad::digamma

fvar< T > digamma(const fvar< T > &x)

**Definition:** digamma.hpp:16

vv\_vari.hpp


---

[ Stan Home Page ]

*© 2011–2014,
Stan Development Team.*
